# Oxygenation of the Prefrontal Cortex during Memory Interference

**DOI:** 10.3390/jcm8122055

**Published:** 2019-11-22

**Authors:** Lindsay Crawford, Liye Zou, Paul D. Loprinzi

**Affiliations:** 1 Exercise & Memory Laboratory, Department of Health, Exercise Science and Recreation Management, The University of Mississippi, Oxford, MS 38677, USA; lcrawfor@go.olemiss.edu; 2 Exercise and Mental Health Laboratory, Shenzhen Key Laboratory of Affective and Social Cognitive Science, Shenzhen University, Shenzhen 518060, China

**Keywords:** cognition, cognitive function, memory, pattern separation

## Abstract

Background: Memory interference occurs when information (or memory) to be retrieved is interrupted by competing stimuli. Proactive interference (PI) occurs when previously acquired information interferes with newly acquired information, whereas retroactive interference (RI) occurs when newly acquired information interferes with previously acquired information. In animal paradigms, the prefrontal cortex (PFC) has been shown to help facilitate pattern separation, and ultimately, attenuate memory interference. Research evaluating the role of the PFC on memory interference among humans is, however, limited. The present study evaluated the relationship between PFC oxygenation on memory interference among humans, with the null hypothesis being that there is no association between PFC oxygenation and memory interference. Methods: A total of 74 participants (M_age_ = 20.8 years) completed the study. Participants completed a computerized memory interference task using the AB-DE AC-FG paradigm, with PFC oxyhemoglobin levels measured via functional near-infrared spectroscopy. Results: For PI, the change in oxygenated hemoglobin for encoding list 1 and retrieval of list 1 showed moderate evidence for the null hypothesis (BF_01_ = 4.05 and 3.28, respectively). For RI, the Bayesian analysis also established moderate evidence for the null hypothesis across all memory task time points. Conclusion: Our study demonstrates evidence of the null hypothesis regarding the relationship between PFC oxygenation and memory interference. Future work should continue to investigate this topic to identify mechanistic correlates of memory interference.

## 1. Introduction

Memory interference, among other factors (e.g., engram trace decay), impairs memory retention. Interference occurs when information (or memory) to be retrieved is interrupted by competing stimuli. There are two main types of memory interference, namely proactive and retroactive. Proactive interference (PI) occurs when previously acquired information interferes with the newly acquired information. Retroactive interference (RI) occurs when the newly acquired information interferes with previously acquired information. In other words, PI occurs when the competing stimuli precede the target information to be learned, whereas RI occurs when the competing stimuli comes after the material to be learned [1]. Interference is strongly based on the similarity of the information; the stronger the similarity between stimuli, the more likely interference is to occur [2].

Memory interference (MI) is typically measured in laboratory settings with paired associative learning tasks [3]. Research participants are exposed to two separate lists of related or unrelated word pairs (e.g., Flower—Desk) and asked to recall them. Common paradigms include AB-CD, AB-AC, AB-ABr, and AB-DE AC-FG. In the present study, we utilized the AB-DE AC-FG approach, as it allows PI and RI to be measured simultaneously. The AB-DE AC-FG method involves exposure to and recall of four separate lists of word pairs (list AB, DE, AC, and FG). List AB and list AC have repeating “A” words (e.g., AB “Sugar”—Temple, AC “Sugar”—Canoe) and the remainder of the lists contain non-repeating words.

The prefrontal cortex (PFC) plays a significant role in the encoding and retrieval of memory [4]. Per the hemispheric encoding/retrieval asymmetry model [5], neuroimaging studies demonstrate that left PFC activation occurs during memory encoding, while right PFC activation occurs during the retrieval of episodic memory [6]. Further, as discussed elsewhere [7], the PFC (particularly the medial PFC) and hippocampus work in concert to facilitate memory formation and retrieval. The hippocampus is viewed as forming and retrieving specific memories, while the PFC accumulates features of related memories that compose the ‘context’ of a set of connected experiences, such as a list in which a set of words appeared [7]. As demonstrated by Guise and Shapiro [8], PFC dysfunction increases the rate of PI, suggesting that optimal PFC functioning may play a protective role against PI [8]. Specifically, they showed that inactivation of the mPFC inhibited hippocampal encoding. Additionally, they demonstrated that increased mPFC activity increased hippocampal activity during learning, which associated with the animal’s ability to adapt to changing rules. Collectively, these findings suggest that the mPFC teaches the hippocampus how to retrieve distinct information from a similar context (e.g., AB-DE AC-FG paradigm), which is also supported by other work [9]. Further, under certain circumstances, the dorsolateral PFC may also help facilitate pattern separation [10]. Similarly, the ventrolateral regions of the PFC have been demonstrated to assist with the ability to select goal-relevant information—activation of this region can strengthen the representation of target information [11,12].

The PFC plays a very active role in guiding memory retrieval by using the relevant context to resolve conflicting information in the retrieval of related and competing memories [7]. However, there is a limited understanding of the neural mechanisms of RI, which underscores the importance of evaluating mechanisms influencing both PI and RI.

Functional magnetic resonance imaging (fMRI) has been used for decades to identify which regions of the brain are activated during cognitive engagement. This technique relies on the temporal coupling of cerebral blood flow and neuronal activation [13]. For this study, we aimed to measure the activation of the PFC during a memory interference task through a relatively newer methodology, namely functional near-infrared spectroscopy (fNIRS) [14]. Skin, bone, and tissue are transparent under near-infrared light, while (oxygenated) hemoglobin and deoxygenated-hemoglobin absorb the light. The fNIRS system measures the density of hemoglobin and deoxygenated-hemoglobin based on the amount of light absorbed, allowing for quantification of the density changes that may occur. This measurement indirectly reflects the functional activities of the brain, with cerebral blood flow (CBF) increasing proportionally to neural activity [15]. This indirect assessment of brain activity may provide useful information into how activation of the PFC affects the degree of memory interference while potentially being more accessible when compared to fMRI or other neuroimaging techniques [11,12,15,16]. 

Research demonstrates that oxygen saturation is associated with memory performance on tasks not inducing a memory interference effect [17]. Based on this information, we hypothesized an inverse association between PFC oxygenation and memory interference, as in, when PFC oxygenation is increased, memory performance would also increase, signifying a decrease in memory interference.

## 2. Methods

### 2.1. Study Design

A total of 74 participants completed the study. Participants completed a computerized memory interference task using the AB-DE AC-FG paradigm while wearing an fNIRS device. The outcome measure for the fNIRS was oxyhemoglobin (HbO_2_) levels. This study was approved by the ethics committee at the University of Mississippi (#19-065; approved on 11 January 2019). All participants provided written consent prior to participation.

### 2.2. Participants

Participants (*N* = 74), age 18–35 years, were selected using a convenience, non-random based sampling approach (e.g., classroom announcement, word of mouth) from the student population at the University of Mississippi. Participants completed a single lab visit lasting approximately 45 min. Informed consent and exclusionary criteria forms were completed by the participant before the experiment commenced. Exclusionary criteria included no exercise 5 h prior and no caffeine 3 h prior to laboratory visit, no illicit drug use in the past 30 days, no concussion in the past 30 days, self-report as a non-daily smoker, and no heavy drinkers (1+ alcoholic drink/day for females or 2+ alcoholic drinks/day for males). These parameters were selected as they may confound memory performance. Demographic information was collected, including age, gender, race/ethnicity, mood-regulating drugs prescriptions, ADD/ADHD diagnosis, and measured height and weight. After demographic information was collected, participants completed the Positive and Negative Affect Schedule (PANAS) to determine their mood state at the moment of their laboratory visit. This mood assessment was assessed as mood has been shown to associate with memory performance [18].

### 2.3. Measurements

#### 2.3.1. fNIRS: Prefrontal Cortex Cerebral Oxygenation

During the memory interference protocol, as well as during a baseline period at rest, cerebral oxygen of the PFC was assessed using functional near-infrared spectroscopy (fNRIS; Oxymon, Artinis, Netherlands). The eight-lead Optode headset (2 × 4 channel, 20 × 7 cm headband, optodes 35 mm apart) was worn on the forehead, just above the supraorbital ridge. The Beer–Lambert law was used to calculate micromolar changes in tissue oxygenation by changes in the concentration of oxyhemoglobin. The outcome measure, HbO_2_, was summed across all eight optode leads and was collected during rest, encoding of list 1, recall of list 1, encoding of list 2, recall of list 2, and the modified modified free recall (details described below). Notably, other outcomes (e.g., deoxyhemoglobin or the difference between oxy- and deoxy-hemoglobin) were evaluated, but their results were similar to the results when restricted to HbO_2_. The time for each time point was approximately as follows: baseline assessment (20 s), encoding of list 1 (50 s), break for 20 s, recall of list 1 (90 s), break for 20 s, encoding of list 2 (50 s), recall of list 2 (90 s), and MMFR (90 s). Through the Artinis software, a Gaussian filter was applied to the data after collection.

#### 2.3.2. Memory Interference

While wearing the fNIRS, the participant completed a memory interference task using the AB-DE AC-FG paradigm. Participants were exposed to list 1 (AB-DE), which consisted of eight word-pairs (e.g., Child—Ticket). After encoding list 1, participants completed a 20 s distractor task where they completed simple arithmetic problems (e.g., (5 × 5) + 11). After the 20 s, participants were then exposed to the Cued Recall list 1 where they were asked to recall the missing word from the target word-pair (e.g., Child—_____). To minimize fNIRS interference, participants did not verbalize their responses, but instead, they wrote them down on paper that was provided to them. Participants completed a 20 s distractor task and were then exposed to list 2 (AC-FG), which consisted of eight word-pairs with some overlap of A words (e.g., Child—Ticket and Child—Letter). After another 20 s distractor arithmetic task, participants were exposed to the Cued Recall of list 2 and attempted to recall the missing word from the word pair. Lastly, a final 20 s distractor task was implemented, and participants completed the modified modified free recall (MMFR) word list where they were asked to recall a combined list of list 1 and list 2. The MMFR consists of all the previously learned word-pairs, where some have only one missing word (DE, FG) and others have two missing word-pair associations (AB, AC) (e.g., Table—Street, Child—Ticket and Letter).

The number of correctly recalled words from list 1 and list 2 were summed individually. Using the participant’s MMFR results, the number of correctly recalled words from the subset of words (AB and DE within list 1 and AC and FG within list 2) was calculated. To calculate this, refer to Table 1 to identify the subset of word pairs (e.g., AB—Beaver Velvet, DE—Circle Meadow, AC—Beaver Parcel, and FG—Salad Pony). The percentage was also calculated by dividing the number correct by four, the total number of word pairs in the subset. Using the cued recall results from list 1 and 2, PI was measured by subtracting the percentage of correctly recalled FG pairs from the percentage of AC pairs (e.g., AC—FG) and RI by subtracting the percentage of correctly recalled DE pairs from the percentage of AB pairs (e.g., AB—DE).

HOW TO SCORE OUTCOMES:

- Score list 1 cued recall. List the number of correctly recalled words (out of 8 total possible).
- Score list 2 cued recall. List the number of correctly recalled words (out of 8 total possible).
  (a) Grade MMFR; break down the list into the AB, DE, AC, FG word-pairs and list the number correct out of 4 total possible for each subset.
  (b) Calculate Proactive Interference (PI) by subtracting the control word-pairs from the interference word-pairs from list 2.
    - AC—FG = PI
  (c) Calculate Retroactive Interference (RI) by subtracting the control word-pairs from the interference word-pairs from list 1.
    - AB—DE = RI

AB and AC are interference pairs, DE and FG are control word-pairs. If an individual performs better on control words (which can be expected), their interference score will be negative, meaning they experienced interference. A positive interference score indicates that the individual experienced less interference and therefore they performed better on the interference word-pairs. For each Study Set (1 and 2), noun-pairs were presented on the computer monitor screen for 5 s each. For each cued-recall test (cued recall 1, cued recall 2, and MMFR), participants had 10 s to complete the recall.

### 2.4. Statistical Analysis

Statistical analyses were completed in JASP (v. 0.9.2; Amsterdam, The Netherlands) statistical software. Both frequentist and Bayesian analyses were performed. For the frequentist analyses, linear regression was performed to examine the relationship between change in cerebral oxygenation and memory interference (PI and RI) during the memory task. A change score for oxyhemoglobin was calculated for each time point within the memory task (i.e., encoding of list 1, retrieval of list 1, encoding of list 2, retrieval of list 2, MMFR) by subtracting the baseline value for oxygenated hemoglobin from the specific time point values (e.g., encoding of list 1 HbO_2_—baseline HbO_2_ = change score). Additional sensitivity analyses included various covariates (i.e., age, gender, BMI, and mood state) in the linear regression models. For the frequentist analyses, statistical significance was set at an a-priori alpha level of 0.05. These frequentist analyses were supplemented with Bayesian linear regression models. Bayesian models were computed because frequentist analyses tell us nothing about whether our hypothesis aligns with our observed data, but rather, informs us of the probability of observing our data assuming the null hypothesis is true. As such, we complemented the frequentist analyses with Bayesian analyses, which informs us of the extent to which our data provides evidence of the alternative or null hypothesis. For the Bayesian linear regression, utilizing a noninformative default prior, Bayes Factors (BF) are reported with a result greater than 3 indicating moderate support for the null hypothesis [19,20,21].

## 3. Results

Descriptive statistics are shown in Table 2. The sample (*N* = 74) was predominately female (72%), non-Hispanic White (65%), and had a mean age of 20.8 years, with a mean BMI of 26.9 kg/m^2^.

Results from the frequentist and Bayesian regression analyses are shown in Table 3. The frequentist linear regression demonstrated that there were no statistically significant relationships between the change scores of oxygenated hemoglobin in PI, nor RI, across the memory task.

The Bayesian analyses demonstrated similar effects. For PI, the change in oxygenated hemoglobin during the time periods of encoding of list 1 and the retrieval of list 1 showed moderate evidence for the null hypothesis (BF_01_ = 4.05 and 3.28, respectively). However, the change in HbO_2_ during the time periods of the encoding of list 2, retrieval of list 2, and retrieval of MMFR demonstrated anecdotal evidence for the null (BF_01_ = 2.91, 2.56, and 1.28, respectively). For RI, the Bayesian analysis established moderate evidence for the null hypothesis across all memory task time periods (Table 3).

We also computed additional sensitivity analyses that evaluated the association between PFC oxyhemoglobin and memory interference across distinct regions of the PFC. That is, we evaluated the relationship between oxyhemoglobin and memory interference (both PI and RI) across the four leads on the right PFC and the four leads on the left PFC. These results were similar to our results displayed in Table 3 (i.e., no association between PFC oxyhemoglobin and memory interference).

## 4. Discussion

The purpose of this experiment was to evaluate the relationship between oxygenation of the PFC and memory interference during a paired-associative memory interference task. We hypothesized that PFC oxyhemoglobin would be positively associated with memory performance (i.e., less memory interference). However, based on our results, our hypothesis was not substantiated.

Oxyhemoglobin levels in the PFC were not associated with memory interference, as we had previously hypothesized. This may be due to multiple factors. For example, there are brain regions implicated in memory interference other than the PFC, such as the hippocampus and amygdala, which were not measured in this experiment. For example, communication between the amygdala and the hippocampus help to facilitate pattern separation of memories or events that have an emotional context to them [22]. The hippocampus may specifically help to facilitate temporal pattern separation, while the amygdala influences the strength of the memory [22,23]. Coordinated theta wave oscillations between the amygdala and hippocampus are thought to induce pattern separation of emotional information. Within the hippocampus, the dentate gyrus functions as a pattern separator by helping to de-correlate inputs [24,25].

In addition to other brain regions being implicated in memory interference resolution, fNIRS may not be sensitive enough to detect a relationship with memory interference as it is only suited for examining global levels of oxygenation within broad regions of the PFC. It cannot isolate specific neurons that are implicated in the neural networks associated with memory interference attenuation. As such, future studies should consider examining brain activity at a more invasive level, which, of course, is less feasible in human studies. This study also utilized a memory interference paradigm with relatively few word pairs, and as such, future work should consider refining this approach (e.g., increase the number of word pairs) to help facilitate a stronger interference effect. Further, our sample was also limited to a non-random sample of students, which may limit the external validity of our study.

Perhaps the discrepancy between our finding and those of others that implicate the PFC in memory interference is due to variations in the population studied and instrumentation utilized. Although speculative, perhaps there is greater accuracy in isolating (e.g., via optogenetics) specific brain regions implicated in MI among non-human models. Further, additional work should evaluate whether the reliability/validity of MI instrumentation varies across populations, which could be contributing to population differences.

Despite these potential limitations, the present experiment has several notable strengths. We investigated a novel study, employed a relatively large sample (*N* = 74) and utilized Bayesian analyses in order to inform us of the extent to which our data provides evidence of the null hypothesis. It would be advantageous for future studies to manipulate PFC oxygenation, a deficiency in the amount of oxygen reaching cerebral tissues, in order to examine how direct manipulation of oxygenation affects memory interference. Previous literature has demonstrated that hypoxia can impair central executive and non-executive cognitive tasks [26]. 

In summary, our study demonstrates evidence of the null hypothesis regarding the relationship between PFC oxygenation and memory interference. Future work should continue to investigate this topic to identify mechanistic correlates of memory interference.

## Figures and Tables

**Table 1:** Illustrative example of the memory interference protocol, including the calculation of proactive and retroactive memory interference.

Study Set 1: AB, DE	Cued Recall 1: A__, D__	Study Set 2: AC, FG	Cued Recall 2: A__, F__	MMFR: A__ __ D__ __ F__ __
WHISKEY STAIR	COFFEE ________	HOTEL TEACHER	**DRIVER** _______	TABLE ____ ____
**TRUCK PENCIL**	**DRIVER** ________	**CHILD LETTER**	DOCTOR ______	HOTEL ____ ____
**CHILD TICKET**	**CHILD** ________	PENNY BROTHER	WINDOW ______	**CHILD** ____ ____
TABLE STREET	SUPPER ________	**TRUCK TOAST**	**SLEEVE** _______	WINDOW ____ ____
**DRIVER SALAD**	**SLEEVE** ________	WINDOW MONEY	HOTEL _______	SUPPER ____ ____
**SLEEVE COLLEGE**	WHISKEY _______	DOCTOR KETTLE	**TRUCK** _______	**TRUCK** ___ ___
COFFEE PANTS	TABLE ________	**SLEEVE BRUSH**	PENNY _______	PENNY ____ ____
SUPPER RECORD	**TRUCK** ______	**DRIVER CANDY**	**CHILD** ______	**SLEEVE** ____ _____
				COFFEE ____ ____
				DOCTOR ____ ____
				**DRIVER** ____ ____
				WHISKEY ____ ____

The bold words represent the interference word pairs (i.e., AB, AC), non-bold words represent the control word pairs (i.e., DE, FG).

**Table 2:** Descriptive statistics of sample (*N* = 74).

Variable	Point Estimate	SD
Gender, % female	72	
Age, mean yrs.	20.8	1.8
Race, % Non-Hispanic White	65	
% Non-Hispanic Black	26	
% Other	1	
BMI, mean kg/m^2^	26.9	6.2
Average min/week of exercise	200.8	79.8

BMI, body mass index.

**Table 3:** Regression results for memory interference and HbO_2_ across the memory task (*N* = 74).

	**Proactive Interference (PI)**						
	**Frequentist Analysis**				**Bayesian Analysis**		
**Model ^†^**	**b**	**95% CI**°	***p*-Value**	***R*^2^**	**Coefficient (95% CI°)**	**Bayes Factor (BF_01_)**	**Evidence Level**
Δ Encoding 1	0.012	−0.128, 0.151	0.87	0.09	−0.002 (−0.088, 0.035)	4.05	Moderate for H_0_
Δ Encoding 2	−0.028	−0.159, 0.103	0.67	0.09	−0.010 (−0.119, 0.013)	2.91	Anecdotal for H_0_
Δ Retrieval 1	−0.021	−0.153, 0.112	0.76	0.09	−0.008 (−0.107, 0.036)	3.28	Moderate for H_0_
Δ Retrieval 2	−0.035	−0.152, 0.082	0.55	0.10	−0.012 (−0.115, 0.014)	2.56	Anecdotal for H_0_
Δ MMFR	−0.070	−0.181, 0.041	0.21	0.11	−0.029 (−0.144, 0.000)	1.28	Anecdotal for H_0_
	**Retroactive Interference (RI)**						
**Model ^†^**	**b**	**95% CI**°	***p*-Value**	***R*^2^**	**Coefficient (95% CI°)**	**Bayes Factor (BF_01_)**	**Evidence Level**
Δ Encoding 1	0.033	−0.104, 0.169	0.64	0.08	0.001 (−0.009, 0.025)	3.71	Moderate for H_0_
Δ Encoding 2	−0.034	−0.163, 0.094	0.60	0.08	−0.002 (−0.024, 0.013)	3.75	Moderate for H_0_
Δ Retrieval 1	−0.001	−0.131, 0.129	0.99	0.07	−2.677 × 10^−4^ (−0.015, 0.018)	4.15	Moderate for H_0_
Δ Retrieval 2	−0.027	−0.141, 0.088	0.65	0.08	−0.001 (−0.025, 0.009)	3.64	Moderate for H_0_
Δ MMFR	0.004	−0.107, 0.114	0.95	0.07	−1.918 × 10^−4^ (−0.014, 0.014)	4.15	Moderate for H_0_

^†^ Each frequentist model was adjusted for age, gender, BMI, and PANAS positive and negative scores. °CI, credible interval. b, change score coefficient.
